# Thalassemic DNA-Containing Red Blood Cells Are under Oxidative Stress

**DOI:** 10.1155/2012/943974

**Published:** 2012-03-04

**Authors:** Mutaz Dana, Eugenia Prus, Eitan Fibach

**Affiliations:** Department of Hematology, Hadassah-Hebrew University Medical Center, Ein-Kerem, P.O. Box 12000, Jerusalem 91120, Israel

## Abstract

We studied the nature of enucleated RBCs containing DNA remnants, Howell-Jolly (HJ) RBCs and reticulocytes (retics), that are characteristically present in the circulation of thalassemic patients, especially after splenectomy. Using flow cytometry methodology, we measured oxidative status parameters of these cells in patients with *β*-thalassemia. In each patient studied, these cells had higher content of reactive oxygen species and exposed phosphatidylserine compared with their DNA-free counterparts. These results suggest that oxidative stress in thalassemic developing erythroid precursors might, through DNA-breakage, generate HJ-retics and HJ-RBCs and that oxidative stress-induced externalization of phosphatidylserine is involved in the removal of these cells from the circulation by the spleen, a mechanism similar to that of the removal of senescent RBCs.

## 1. Introduction

The development of red blood cells (RBCs) from their progenitors in the bone marrow includes the process of enucleation in which the final stages of nucleated erythroid precursors (orthochromatic normoblasts) expel their nuclei to generate enucleated reticulocytes (retics), which leave the marrow and mature into RBCs [1]. Normally, nucleated RBCs (normoblasts) are undetectable in the circulation, but in some hematological pathologies (e.g., thalassemia and sickle cell disease) they can be found in large numbers [1]. These diseases are also characterized by mature RBCs and retics that contain DNA remnants, that are called Howell-Jolly (HJ) bodies [1]. The frequency of these cells, which is very low, has been quantified using a flow cytometry technique [2–4].

The spleen is the major site of the reticuloendothelial system where senescent RBCs at the end of their life-span are removed by erythrophagocytosis [5]. It also removes from the circulation normoblasts and HJ-cells; thus, in thalassemia and sickle cell disease, the number of these cells in the patients' circulation increases considerably following splenectomy [1].

The removal of senescent RBCs has been attributed to various mechanisms [5], including exposure (externalization) of phosphatidylserine (PS) on their surface [6]. The macrophages of the reticuloendothelial system carry surface receptors that specifically bind PS, by which they internalize senescent RBCs [7]. The mechanism by which normoblasts and HJ-cells are removed from the circulation is unknown.

We have previously shown that in hemolytic anemias, including thalassemia and sickle cell disease, RBCs are under oxidative stress [8], and they generate more reactive oxygen species (ROS) and contain less reduced glutathione than normal RBCs, which results in membrane changes such as lipid peroxidation and externalization of PS.

Using flow cytometry, in the present study we show that HJ-RBCs and retics are under oxidative stress and carry exposed PS, which may present the trigger for their phagocytosis by macrophage and removal in the spleen.

## 2. Materials and Methods

**Blood Samples**

Peripheral blood (PB) samples were obtained from normal donors and splenectomized and nonsplenectomized patients with *β*-thalassemia intermedia and major. The samples were obtained from the counting vials after all diagnostic laboratory tests were completed. The research was approved by the Hadassah-Hebrew University Medical Centre Human Experimentation Review Board. The patients' mutations and some relevant clinical parameters (e.g., transfusion and chelation therapy, splenectomy) were previously summarized [9]. In polytransfused patients, blood samples were obtained before transfusion, that is, at least 3 weeks following the previous transfusion. Informed consent was obtained in all cases.

**Flow Cytometry Measurements of Oxidative Stress Markers**

Cells were stained for transferrin-receptor by incubating with 5 *μ*L of APC-conjugated antibodies (Ab) to CD71 at 4°C for 30 minutes. The sample was washed and then divided into two aliquots: one aliquot was stained for ROS with 2′-7′-dichlorofluorescin diacetate (DCFH, Sigma, St, Louis, MO), at final concentration of 0.1 mM, at 37°C for 15 minutes, then washed three times with Ca^++^- and Mg^++^-free Dulbecco's phosphate-buffered-saline (PBS) (Biological Industries, Beit-HaEmek, Israel). A stock solution of 20 mM DCF was prepared in methanol (Bio Lab, Jerusalem, Israel). The other aliquot was stained for external phosphatidylserine (PS), by suspending the cells in 100 *μ*L of calcium buffer ((10 mM HEPES, 140 mM NaCl and 2.5 mM CaCl_2_ (pH 7.4)) and 2 *μ*L of FITC-conjugated Annexin-V (IQ Products, Groningen, The Netherlands). After 15 minutes at room temperature, in the dark, the cells were washed three times with calcium buffer and resuspended in 0.5 mL of the same buffer.

For every assay, 2 *μ*L of propidium iodide (PI, Mallinckrodt Chemical Works, St. Louis, MO), dissolved in 0.1% sodium citrate, was added before analysis. Cells stained with anti-CD71 Ab alone, cells stained with anti-CD71 Ab and annexin-V, or cells stained with anti-CD71 Ab and DCF were used as controls to set the compensation levels. Following treatment as indicated above, the cells were analyzed with a Fluorescence Activated Cell Sorter (FACS-calibur, Becton-Dickinson, Immunofluorometry systems, Mountain View, CA). Instrument calibration and settings were performed using CaliBRITE-3 beads (Becton-Dickinson). The cells were passed at a rate of ~1,000 per second, using saline as the sheath fluid. A 488 nm argon laser beam was used for excitation. Threshold was set on forward light scatter (FSC) to exclude platelets and cell debris. Gates were set on RBCs, HJ-RBCs, retics, HJ-retics, normoblasts, and WBCs. Cells labeled with DCF and annexin-V were detected by the FL-1 PMT, and cells labeled with APC-conjugated anti-CD-71 Ab and PI were detected by the FL-4 and FL-2 PMT, respectively. All PMTs were set on log amplification. The Mean Fluorescence Intensities (MFIs) and the percentages of positive cells were calculated using the FACS-equipped CellQuest software (Becton-Dickinson). The results are expressed as the average ± standard deviation (SD) and compared using the two-sample Student's *t*-test for differences in means.

## 3. Results and Discussion

**Figure 1:**
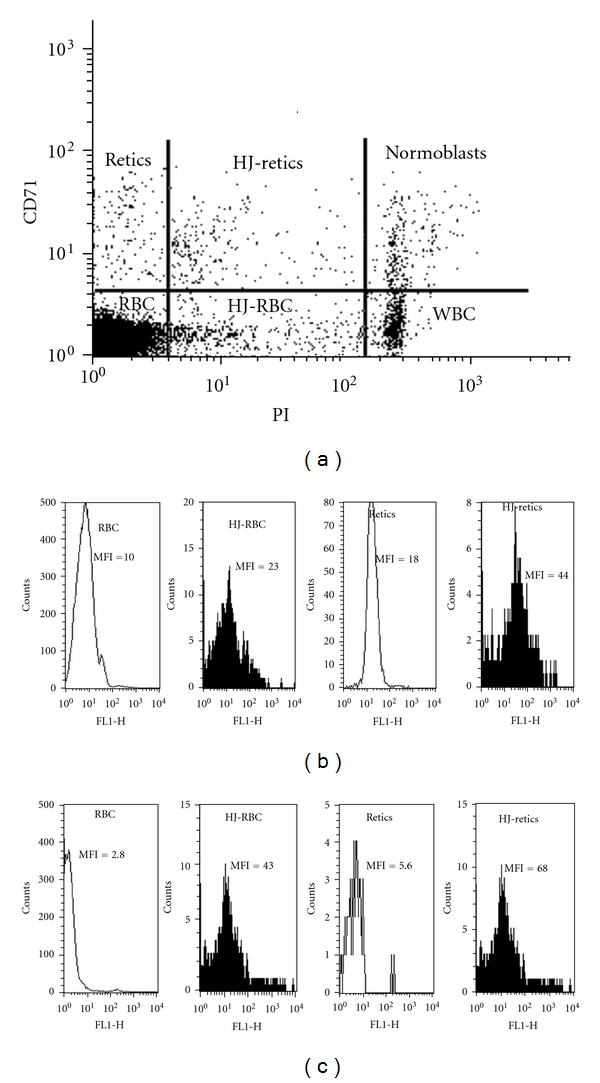
Flow cytometry analysis of ROS and PS in blood cells. Blood cells from a splenectomized *β*-thalassemic patient were simultaneously stained with an anti-CD71 antibody and propidium iodide (PI), and either DCF for measurement of ROS or annexin-V for measurement of external PS. (a) A CD71 versus PI dot-plot identifying cells as RBCs (CD71-PI-), HJ-RBCs (CD71-PI+), WBCs (CD71-PI++), retics (CD71+PI-), HJ-retics (CD71+PI+), and normoblasts (CD71+PI++). ((b)-(c)) Fluorescence distribution histograms of each cell population with respect to ROS (b) and PS (c). The results expressed as the mean fluorescence index (MFI) are presented for each cell population.

PB cells were simultaneously stained with an anti-CD71 Ab and PI, and either DCF or annexin-V. The anti-CD71 Ab marks the transferrin receptor, and PI the nucleic acid content. To evaluate the contribution of RNA (particularly in retics which contain small amounts of residual RNA) to the PI staining, PB cells were stained with PI in the presence or absence of RNase (0.4 mg/mL, Invitrogen, Carlsbad, CA). No difference was noted in the pattern of PI staining between these samples. The staining procedure identified cells as RBCs (CD71-PI-), HJ-RBCs (CD71-PI+), WBCs (CD71-PI++), retics (CD71+PI-), HJ-retics (CD71+PI+), and normoblasts (CD71+PI++).  Figure 1(a) shows a flow cytometry dot-plot (PI versus CD71) analysis of a blood sample derived from a representative splenectomized *β*-thalassemic patient, indicating the various cell populations. The fluorescence distribution histograms of each cell population with respect to DCF-fluorescence, indicating generation of ROS, and annexin V-fluorescence, indicating exposed PS, with their MFIs, are shown in Figures 1(b) and 1(c), respectively. The results indicate higher ROS and PS in retics than in mature RBCs, and, more critically, in HJ-cells compared with their non-HJ counterparts: in the experiment presented in Figure 1(b), showing ROS results, the MFI of HJ-RBCs was 2.3-fold higher than that of RBCs, and the MFI of HJ-retics was 2.4-fold higher than retics. In Figure 1(c), showing PS results, the MFI of HJ-RBCs was 15.3-fold higher than that of RBCs, and the MFI of HJ-retics was 12.1-fold higher than retics.

**Figure 2:**
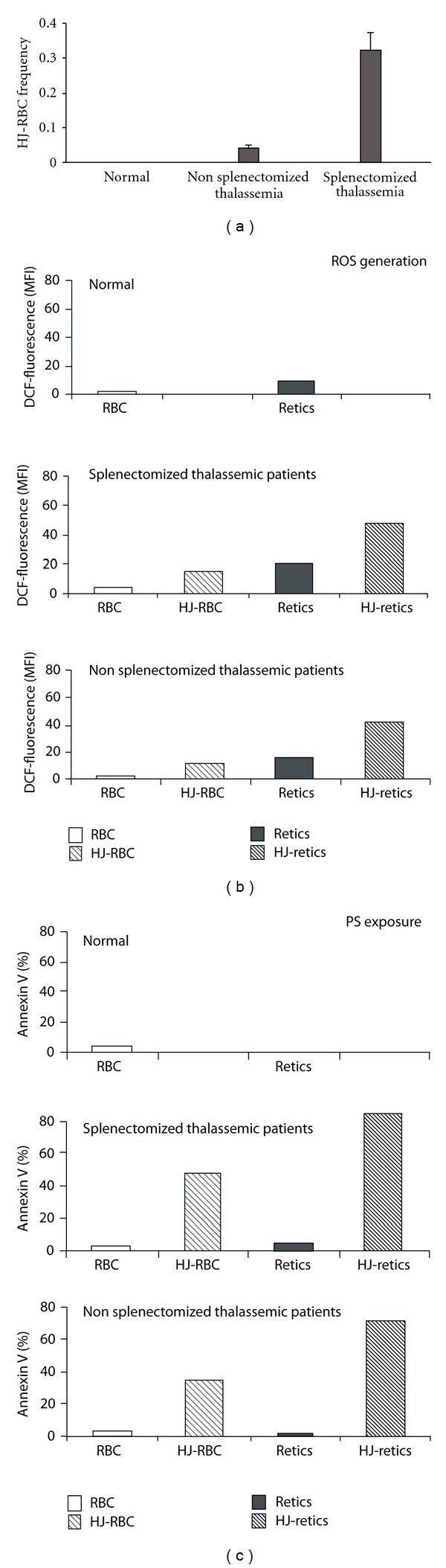
The frequency of HJ-cells and their oxidative status in normal donors and thalassemic patients. Cells obtained from the blood of normal donors and splenectomized and nonsplenectomized thalassemic patients (*N* = 6 in each group) were stained and analyzed as in legends to Figure 1(a). (a) The frequency of HJ-RBCs. (b) ROS generation. (c) PS exposure. The results are expressed as the percentage in the RBC population (a), the average ± S.D of the mean DCF-fluorescence index (MFI) for ROS (b) and the percentage of cells positively stained with annexin-V for PS (c).

Figure 2(a) depicts the frequency of HJ-RBCs in the PB of normal donors and in thalassemic patients. The results show no HJ-RBCs in normal donors and much higher frequency of HJ-RBCs in splenectomized patients compared with nonsplenectomized patients. Figures 2(b)-2(c), which summarize the average ROS generation and percentage of PS-exposing cells, show that both parameters were significantly higher in HJ-RBCs versus RBCs and in HJ-retics versus retics. The results also show that both parameters are higher in cells from splenectomized versus nonsplenectomized patients, suggesting that the spleen removes the most damaged cells.

Although the process of nuclear expulsion from developing RBC precursors has been studied extensively [10, 11], the reasons for nuclear remnants (HJ-bodies) leftover in enucleated retics and RBCs in certain diseases have not been studied before. We now report that in *β*-thalassemia the generation of ROS and the externalization of PS, both parameters of oxidative stress, are elevated in HJ-retics and HJ-RBCs compared with their no-HJ-containing counterparts. ROS may be the cause of HJ formation. They are known to cause DNA breaks [12] that may generate micronuclei in various cell types [13], including lymphocytes and neutrophils. The occurrence of micronuclei has been used as a biomarker for cytogenetic damage [14, 15]. These micronuclei are equivalent to the HJ bodies in RBCs. The mechanism of HJ bodies' formation must occur prior to nuclear expulsion. We have previously demonstrated that thalassemic erythroid precursors, including orthochromatic normoblasts, are at higher oxidative status than their normal counterparts [9]. It might be hypothesized that DNA/nuclear breaks induced by oxidative stress might result in incomplete expulsion of the nuclear material, resulting in nuclear remnants which remain in retics and mature RBCs.

Several studies [16], including our own [9], indicated that ROS stimulate PS externalization on RBCs. Exposed PS was suggested, in addition to other mechanisms such as reduced expression of CD47 [17] and binding of autologous immunoglobulins and opsonins [18, 19] to signal erythrophagocytosis and removal of senescent RBCs from the circulation. To our knowledge, the signals for phagocytosis and removal of peripheral blood normoblasts or HJ-cells have not been studied. Our findings of enhanced exposure of PS on HJ-cells might suggest that exposed PS might participate in the removal of such cells by the spleen, although other signals cannot be ruled out.

In conclusion, the results of the present study suggest that oxidative stress in developing erythroid precursors might generate HJ-retics and HJ-RBCs and that oxidative stress-induced externalization of PS might be involved in their removal from the circulation by the spleen, a mechanism similar to that of the removal of aging (senescent) RBCs.
